# Stress-dose hydrocortisone reduces critical illness-related corticosteroid insufficiency associated with severe traumatic brain injury in rats

**DOI:** 10.1186/cc13067

**Published:** 2013-10-16

**Authors:** Xin Chen, Zilong Zhao, Yan Chai, Lanlan Luo, Rongcai Jiang, Jingfei Dong, Jianning Zhang

**Affiliations:** 1 Department of Neurosurgery, Tianjin Medical University General Hospital, Tianjin 300052, P.R. China; 2 Tianjin Neurological Institute, Tianjin 300052, P.R. China; 3 Key Laboratory of Post-trauma Neuro-repair and Regeneration in Central Nervous System, Ministry of Education, Tianjin 300052, P.R. China; 4 Tianjin Key Laboratory of Injuries, Variations and Regeneration of Nervous System, Tianjin 300052, P.R. China; 5 Department of Psychology, Tianjin Huanhu Hospital, Tianjin 300060, P.R. China; 6 Hematology Division, Department of Medicine, Puget Sound Blood Research Institute (JD), University of Washington, Seattle, WA 98104, USA

## Abstract

**Introduction:**

The spectrum of critical illness-related corticosteroid insufficiency (CIRCI) in severe traumatic brain injury (TBI) is not fully defined and no effective treatments for TBI-induced CIRCI are available to date. Despite growing interest in the use of stress-dose hydrocortisone as a potential therapy for CIRCI, there remains a paucity of data regarding its benefits following severe TBI. This study was designed to investigate the effects of stress-dose hydrocortisone on CIRCI development and neurological outcomes in a rat model of severe traumatic brain injury.

**Methods:**

Rats were subjected to lateral fluid percussion injury of 3.2-3.5 atmosphere. These rats were then treated with either a stress-dose hydrocortisone (HC, 3 mg/kg/d for 5 days, 1.5 mg/kg on day 6, and 0.75 mg on day 7), a low-dose methylprednisolone (MP, 1 mg/kg/d for 5 days, 0.5 mg/kg on day 6, and 0.25 mg on day 7) or control saline solution intraperitoneally daily for 7 days after injury.

**Results:**

We investigated the effects of stress-dose HC on the mortality, CIRCI occurrence, and neurological deficits using an electrical stimulation test to assess corticosteroid response and modified neurological severity score (mNSS). We also studied pathological changes in the hypothalamus, especially in the paraventricular nuclei (PVN), after stress-dose HC or a low dose of MP was administered, including apoptosis detected by a TUNEL assay, blood–brain barrier (BBB) permeability assessed by brain water content and Evans Blue extravasation into the cerebral parenchyma, and BBB integrity evaluated by CD31 and claudin-5 expression. We made the following observations. First, 70% injured rats developed CIRCI, with a peak incidence on post-injury day 7. The TBI-associated CIRCI was closely correlated with an increased mortality and delayed neurological recovery. Second, post-injury administration of stress-dose HC, but not MP or saline increased corticosteroid response, prevented CIRCI, reduced mortality, and improved neurological function during the first 14 days post injury dosing. Thirdly, these beneficial effects were closely related to improved vascular function by the preservation of tight junctions in surviving endothelial cells, and reduced neural apoptosis in the PVN of hypothalamus.

**Conclusions:**

Our findings indicate that post-injury administration of stress-dose HC, but not MP reduces CIRCI and improves neurological recovery. These improvements are associated with reducing the damage to the tight junction of vascular endothelial cells and blocking neuronal apoptosis in the PVN of the hypothalamus.

## Introduction

Traumatic brain injury (TBI) remains a leading cause of death and disability among adolescent males and young adults in China, with an estimated annual cost of $20 billion for medical expenses. Approximately 1 million TBI cases are reported each year in emergency rooms throughout China, resulting in 100,000 deaths annually [1].

Critical illness-related corticosteroid insufficiency (CIRCI), defined by the American College of Critical Care Medicine, is used to describe dysfunctions of the hypothalamic-pituitary-adrenal (HPA) axis that occurs during critical illness [2]. It is characterized by an exaggerated and protracted pro-inflammatory response and corticosteroid resistance, leading to an inadequate corticosteroid response to severe stress [3]. The incidence of CIRCI varies considerably (up to 77%) in patients with sepsis, shock, acute respiratory distress syndrome (ARDS), and severe pancreatitis [4-8]. Results from recent clinical trials demonstrate that CIRCI is found in 50 to 70% of trauma patients, with approximately 34% mortality despite therapy [9]. However, the incidence of CIRCI in severe TBI has not been fully defined [10].

The pathogenesis of CIRCI in the acute phase of TBI has characteristic features that distinctively differ from those found in other severe illness [11,12]. We have previously used electrical stimulation tests, which mimic acute stress that activates the HPA axis in the sub-acute phase of TBI, to assess the adrenal insufficiency in a rat model of TBI. Pathological changes in the model rats are similar in pathophysiology to CIRCI in patients as defined by the American College of Critical Care Medicine [13]. Our previous study in rats suggests that CIRCI could develop during the acute phase of TBI and is closely associated with increased mortality [13]. The apoptosis of cells in the adenohypophysis and in the paraventricular nucleus (PVN) of the hypothalamus might be the pathological characteristics of TBI-associated CIRCI. Additional damage to the HPA axis could further aggravate acute CIRCI, leading to a high fatality [13]. Interventions targeting CIRCI have been implemented in such critical illness [14] as septic shock [15], community-acquired pneumonia [16], and stroke [17], but not in severe TBI, largely because the underlying mechanisms for TBI-associated CIRCI remain poorly understood.

Recent clinical evidence suggests that relatively long-term use of stress-dose of hydrocortisone (HC) (200 mg per day for 7 days), a natural form of steroid hormone in humans, significantly reduces mortality in patients with septic shock and CIRCI without increasing adverse events [15]. More importantly, stress-dose HC has also been shown to decrease the incidence of hospital-acquired pneumonia and the time on mechanical ventilation in patients with polytrauma and CIRCI. It is particularly efficient for polytrauma patients with TBI [14]. Recent experimental data suggest that HC could upregulate metal homeostasis regulator MT-1/2 [18] and anti-apoptotic factor PKCϵ to protect the brain [19]. HC has also been demonstrated to maintain the integrity of endothelial cell tight junctions and to stabilize the blood–brain barrier (BBB) as a mode of glucocorticoid action at a molecular level in the human brain vasculature [20]. However, no data on the use of stress-dose HC to treat CIRCI in severe TBI are available to date. Whether it reduces CIRCI and improves prognosis after severe TBI remains unknown.

Accordingly, we hypothesize that stress-dose of hydrocortisone decreases neural apoptosis and restores BBB function in the hypothalamus, thus boosting the acute corticosteroid response and reducing post-traumatic CIRCI and TBI mortality. We tested this hypothesis in a rat model of experimentally controlled TBI.

## Materials and methods

Animal care and experiments were conducted in accordance with the ethical approvals stipulated by the Small Animal Protection Board of Tianjin Medical University.

### Animals

Male Wistar rats weighing 300 to 350 g at the time of surgery were supplied by Experimental Animal Laboratories of the Academy of Military Medical Sciences (Beijing, China). Rats were housed individually in a temperature-controlled (22°C) and humidity-controlled (60%) condition, and maintained on a standard 12-h light/dark cycle (7:00 a.m. to 7:00 p.m) with access to food and water *ad libitum*. All experimental procedures were performed during the light phase between 10:00 a.m. and 2:00 p.m. at the nadir of the circadian cycle for circulating steroid in rats.

**Figure 1:**
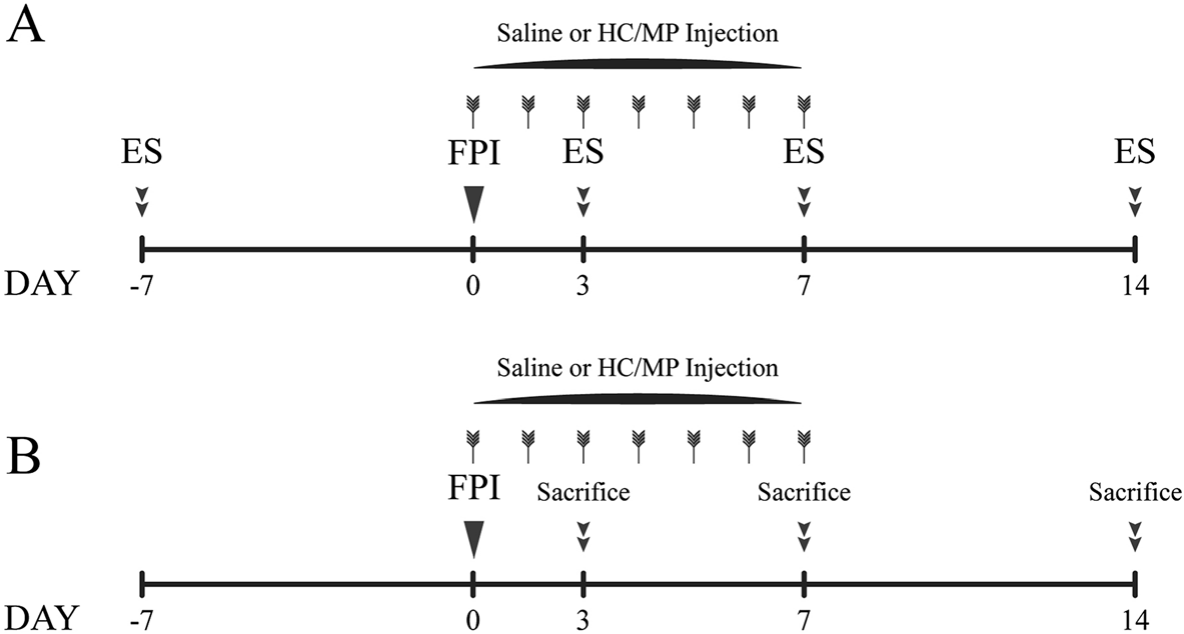
**Study design and experimental procedures. (A)** Part I: to evaluate the effect of stress-dose hydrocortisone (HC) on corticosteroid responses and incident critical illness-related corticosteroid insufficiency after traumatic brain injury; HC or methylprednisolone (MP), or saline in the case of injury controls, was intraperitoneally (i.p.) injected daily from 0 to 7 days after fluid percussion injury (FPI). Electrical stimulation (ES) was administered to 30 rats randomly assigned from each of the four groups: pre-injury day 7, and post-injury day 3, day 7 and day 14. **(B)** Part II: the remaining rats were injected with HC or MP or saline once a day for 7 days after FPI to evaluate the extent of brain edema, BBB integrity and permeability, and apoptosis in the hypothalamal cells; 22 rats from each of the two groups (naïve and normal HC) and 50 from each of the three groups (injury control, low-dose MP, and stress-dose HC) were sacrificed at each time point (post-injury day 3, 7 and 14) to measure brain water content, to detect Evans blue extravasation and expression of CD31 and claudin-5, and to quantify terminal deoxynucleotidyl transferase dUTP nick end labeling-positive cells in the hypothalamus.

Rats were grouped by treatments into: naïve, injury control, HC normal, low-dose methylprednisolone (MP), and stress-dose HC (Table 1). MP, which is a synthetic glucorticoid drug and has also been shown to reduce apoptosis and BBB permeability [21,22], was tested in this setting as the control. The entire experiment consisted of two parts. In the first part, rats from each treatment group were randomly assigned to be evaluated for corticosteroid response and to determine CIRCI incidence after TBI. In the second part, histological examination was performed on the hypothalamus to evaluate brain edema, BBB integrity and permeability, and cellular apoptosis (Figure 1).

**Table 1:** Experimental groups

**Groups**	**Number of rats**	**Treatments**
Naïve	66	None
Hydrocortisone normal treatment	96	Hydrocortisone 0.75 to 3,0 mg/kg
Injury control	227	Anesthesia, surgical procedure, fluid percussion injury and equal volume of saline
Low-dose methylprednisolone treatment	185	Anesthesia, surgical procedure, fluid percussion injury and 0.25 to 1.0 mg/kg methylprednisolone
Stress-dose hydrocortisone treatment	120	Anesthesia, surgical procedure, fluid percussion injury and 0.75 to 3.0 mg/kg hydrocortisone

### Fluid percussion-induced brain injury

Fluid percussion-induced brain injury (FPI) is an extensively characterized and broadly used preclinical model of closed head injury [23,24]. Briefly, rats were anesthetized with a single intraperitoneal injection of chloral hydrate (3.0 ml/kg) and placed in a stereotaxic frame. Craniotomy (4.0 × 4.0 mm) was performed over the right parietal bone, 2.0 mm lateral from the sagittal suture and 2.5 mm caudal from the coronal suture with intact dura mater. They were subjected to FPI of 3.2 to 3.5 atmosphere (atm) 24 h after craniotomy as we have previously described [25].

### Neurological assessment

Neurological functions were assessed at baseline before injury, on post-injury day 1, day 3, day 7, and day 14 using the modified neurological severity score (mNSS) [26]. Assessments in the neuroscore include motor, sensory, reflex, and balance tests. These scores were used 1) to ensure the relative uniformity in injury severity; 2) to compare neurological recovery among rats receiving different treatments. The tests were performed by the same observer who was blinded to the experimental conditions and treatments.

### Glucocorticoid administration

After FPI, rats were on a 7-day regimen of either stress-dose HC (3 mg/kg/d for 5 days, 1.5 mg/kg on day 6, and 0.75 mg on day 7) [14] or low-dose MP (1 mg/kg/d for 5 days, 0.5 mg/kg on day 6, and 0.25 mg on day 7) [27]. As injury controls, naïve rats were given the same dose of HC. For drug controls rats underwent the same surgical procedures and FPI, but received an equal volume of 0.9% saline (Table 1, Figure 1).

### Assessment of corticosteroid response

#### Electrical stimulation

Thirty rats from each of the four experimental groups were examined for corticosteroid response by being subjected to electrical stimulation (ES) on four test days: the seventh day before injury, and post-injury day 3, 7, and 14 (Figure 1) as described by Ji *et al.*[28]. In brief, ear-clip electrodes were placed on both ears of the animal while awake, and were connected to an electrical stimulator (unit J18A1; Quanrikang, Beijing, PR China). An electric current (1s at 90 mA) was applied to induce acute stress. On the days of electrical stimulation, blood was collected pre-ES, and at 30 minutes, 90 minutes, and 24 h post-ES to measure dynamic changes of serum corticosterone (CORT), the most abundant circulating steroid in rats. Blood samples were collected from the orbital sinus under inhaled light anesthesia (enflurane; Abbott, Shanghai, PR China) into a dry centrifuge tube and centrifuged at 3,000 rpm for 8 minutes. Cell-free plasma was collected and stored at -80°C for further CORT assay [29].

#### Serum corticosterone assay and critical illness-related corticosteroid insufficiency assessment

Serum levels of CORT were measured using a commercial ELISA kit (Diagnostic Systems Laboratories, Webster, TX, USA), according to the manufacturer’s instructions. Corticosteroid responses of injured rats were assessed by a CORT increase index (CII), which is the difference between the post-ES peak value and pre-ES baseline value of CORT, divided by the pre-ES baseline value of CORT. A CII value less than 2.5 was classified as acute CIRCI as described previously [13].

### Brain water content

Edema in the hypothalamus was determined by measuring brain water content (BWC) as previously described [30]. Rats were sacrificed with an overdose of chloral hydrate (30 ml/kg, i.p.). The brains were promptly removed, and a thick (5-mm) coronal slice was made from the brain (beginning at the bregma). The hypothalamus surrounding the third ventricular area was removed with a 3-mm punch, and immediately weighed (wet weight) and then placed in an incubator at 100°C for 24 h. The samples were weighed again to determine the dry weight. The BWC was calculated from:

$$\begin{array}{l} \text{BWC}\;\left(\%\right)=\left[\left(\text{Wet}\;\mathrm{Weight}‒\mathrm{Dry}\;\mathrm{Weight}\right)/\text{Wet}\;\text{Weight}\right]\\ \;\times 100\% \end{array}$$

### Permeability of the blood–brain-barrier

BBB permeability of the hypothalamus was assessed by measuring the extravasation of Evans blue (EB) dye. EB dye injected intravenously binds instantaneously to albumin and other plasma proteins and serves as a marker for plasma exudation [31]. In brief, EB (2% in PBS, Sigma) was injected slowly through the jugular vein (4 ml/kg) and allowed to circulate for 1.5 h. Then, rats were sacrificed and transcardially perfused with 1 × PBS followed by 0.9% saline. The brain was removed and the hypothalamus around the third ventricle was obtained as described above. The tissue was frozen in -55°C isopentane and freeze dried. Freeze-dried specimens were homogenized in formamide (1:20) and incubated at 60°C overnight. The homogenate was centrifuged at 14,000 rpm for 30 minutes to collect supernatant. EB content in the supernatant was determined spectrophotometrically at O.D. 620 nm (Thermo Scientific, Waltham, MA, USA).

### Detection of neuronal apoptosis

Terminal deoxynucleotidyl transferase dUTP nick end labeling (TUNEL) is a well-defined method for detecting apoptotic DNA fragmentation in a cell [32]. We used the In Situ Cell Death Detection Kit, POD (Roche, Mannheim, Germany) to detect apoptosis in the PVN of the hypothalamus according to the manufacturer’s instructions. Rats were sacrificed at designated time points with overdose of chloral hydrate (30 ml/kg, i.p., Figure 1). The brain was removed and fixed in 4% paraformaldehyde for 24 h. The tissue was then paraffin embedded and processed for immunohistological examination. Two successive brain sections from each rat were stained with H&E and TUNEL staining, respectively [32]. Briefly, 5-μm coronal sections from the brain (0.7 to 2.3 mm posterior to the bregma) were affixed to poly-L-lysine-coated slides and deparaffinized. The sections were rehydrated and treated with protease K (20 mg/ml in 10 mM Tris–HCl; pH 7.5 to 8.0) for 30 minutes at room temperature. They were then incubated at 37°C with the TUNEL reaction mixture in a humidified chamber for 3.5 h. The reaction was terminated by washing slides with 0.01 M PBS (pH 7.4) and then incubated with Converter-POD (anti-fluorescein antibody conjugated with horseradish peroxidase) for 2.5 h at 37°C, followed by color development at 20°C with 3,3-diaminobenzidine (DAB) substrate. The sections were counterstained with hematoxylin and mounted with neutral balsam.

Apoptotic cells in bilateral areas of the paraventricular nucleus (PVN) in the hypothalamus were counted using light microscopy at 400× (CH20BIMF200; Olympus, Tokyo, Japan). They were quantified by the total number of labeled cells in four sections (40 μm apart).

### Immunohistochemistry and fluorescence intensity quantification

At designated time points, rats were sacrificed with an overdose of chloral hydrate (30 ml/kg, i.p.) and immediately perfused through the heart with the phosphate buffer followed by 2% paraformaldehyde, 10 mM sodium periodate, and 70 mM L-lysine (2% PLP). The brain was dissected out, post-fixed by immersion in the same fixative for 1 h, and incubated in a solution of 30% sucrose overnight. It was embedded in optimum cutting temperature (OCT) medium (Sakura, Torrance, CA, USA) on dry ice, and stored at -80°C. Coronal sections of 10 μm at the hypothalamus level were made on a cryostat at -20°C and imprinted on poly-L-lysine-coated slides. These sections were stained for claudin-5 (a tight-junction marker) and CD31 (an endothelial cell marker).

After air-drying, the sections were fixed with 2% PLP and rinsed three times in PBS (pH 7.4). They were blocked in 1% normal donkey serum in PBS with 0.1% Triton X-100 (PBS/Tween (PBST)) at room temperature for 1 h, followed by incubation with either a rabbit anti-claudin-5 antibody diluted at 1:500 (Abcam Cambridge, MA, USA) or a mouse anti-CD31 antibody diluted 1:500 (Invitrogen Grand Island, NY, USA) in PBST containing 1% normal donkey serum at 4°C overnight following extensive washing in PBS; the sections were incubated with an Alexa Fluor 488-conjugated goat anti-rabbit IgG antibody at a dilution of 1:1,000 (Invitrogen) and an Alexa Fluor 568-conjugated goat anti-mouse IgG antibody (Invitrogen) for 3 h at room temperature. Images of immunofluorescence were captured using an Olympus IX81 microscope (Shinjuku-ku, Tokyo, Japan).

The bilateral area of the PVN in the hypothalamus from each section and three sections from each rat were analyzed. Fluorescence intensities, measured using Image J software (from the National Institutes of Health, Bethesda, MD, USA), were averaged for each section, and the three sections were averaged for each rat.

### Statistical analyses

Data were analyzed using SPSS (SPSS Inc., Chicago, IL, USA). Kaplan-Meier survival analysis with the log-rank significance test was used to measure the mortality rates among rats with different treatments. Data on mNSS, CII, apoptotic cell, BWC, EB extravasation and CD31 and claudin-5 expression were analyzed by analysis of variance (ANOVA) followed by post hoc least significant difference (LSD) test or the Dunnett T3 test, whereas acute CIRCI data were analyzed by the Chi-square test. The Pearson Chi-square test was performed to assess the strength of the relationship between the incidence of acute CIRCI and mortality. A *P*-value <0.05 was considered statistically significant.

## Results

### Baseline characteristics

A total of 694 Wistar rats were studied. Among them, 532 were subjected to severe FPI of 3.2 to 3.5 atm, which results in severe TBI. The rest were naïve rats and rats that received HC without surgery and FPI (Table 1). At an injury level of 3.2 to 3.5 atm, rats lapsed into unconsciousness with apnea, regaining spontaneous breathing within 140 to 160 s. Consciousness returned in approximately 280 to 340 s. Severe disturbances in motor function and balance were observed after injury. The motor dysfunction recovered gradually within 7 days, whereas balance improved more slowly.

### Stress-dose hydrocortisone reduced traumatic brain injury-associated mortality

**Figure 2:**
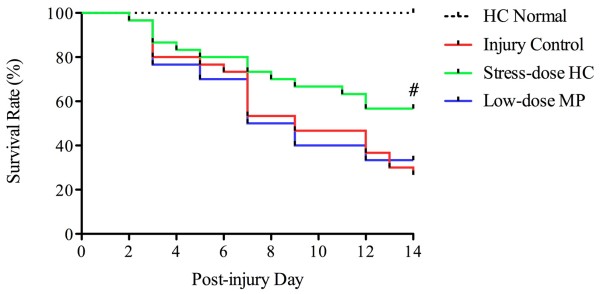
**Survival of rats after traumatic brain injury.** The number of rats treated with stress-dose hydrocortisone (HC) that died during the 14-day post-injury follow-up period was significantly lower compared to those from injury control (summarized in Table 2). The data were from rats that were evaluated for corticosteroid response. Rats used for histological analyses were not included (log-rank test, ^#^Chi-square = 4.344, df = 1, *P* = 0.0371 as compared to injury control). MP, methylprednisolone.

In the first part of the study (Figure 1), 55 rats did not survive after injury and HC or MP treatment (Table 2 and Figure 2). The mortality rate of rats receiving stress-dose HC (43.3%) was significantly lower than those of rats receiving low-dose MP treatment (66.7%) and was lower than in the injury control (73.3%) (Figure 2).

**Table 2:** Mortality of experimental groups

**Groups**	**Outcome**		**Total (number)**
	**Death (number)**	**Survival (number)**	
Hydrocortisone normal treatment	0	30	30
Injury control	22	8	30
Low-dose methylprednisolone treatment	20	10	30
Stress-dose methylprednisolone treatment	13	17	30
Total	55	65	120

### Stress-dose hydrocortisone improved neurological outcomes

**Figure 3:**
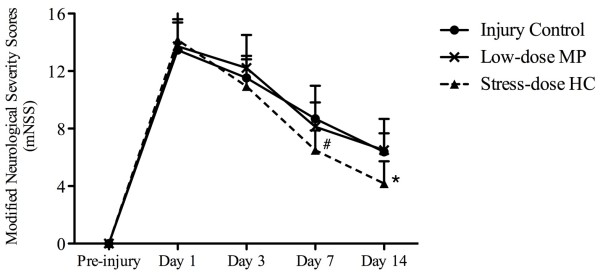
**Effects of stress-dose hydrocortisone on neurological outcomes.** Injured rats treated with stress-dose hydrocortisone (HC) had significantly lower mNSS scores at day 7 and day 14 after traumatic brain injury when compared with the injury control group. Data are presented as means ± SD. Analysis of variance: F_(2,50)_ = 7.208, *P* = 0.002 for day7; post hoc Dunnett T3 test, ^#^*P* = 0.000 for stress-dose HC treatment versus injury control, F_(2,32)_ = 7.762, *P* = 0.002 for day 14; post hoc least significant difference test, *P = 0.006 for stress-dose HC treatment versus injury control. Day 1: n = 30 from each group; day 3: n = 23 from injury control, n = 23 from low-dose methylprednisolone (MP), n = 26 from stress-dose HC; day 7: n = 16 from injury control, n = 15 from low-dose MP, n = 22 from stress-dose HC; day 14: n = 8 from injury control, n = 10 from low-dose MP, n = 17 from stress-dose HC.

We compared mortality among rats from the four experimental groups during a 14-day follow-up period and found no difference in mNSS 24 h after FPI (Figure 3). This indicates that the injury was comparable among rats in all experimental groups. FPI at 3.2 to 3.5 atm over the cortex of the right hemisphere led to neurological deficits measured by mNSS (Figure 3). On post-injury day 1, injured rats presented with high scores on motor, sensory and beam balance tests, as well as absent reflexes and abnormal movements. The recovery of neurological functions began on day 3 after injury and lasted to post-injury day 14, when rats suffered from residual neurological deficiencies, presenting with high scores on sensory and beam balance tests. The mNSS scores for the stress-dose HC treatment were significantly improved on day 7 (mean 6.5 ± SD 1.4; *P* <0.05) and day 14 (4.2 ± 1.6; *P* <0.05) as compared to those for low-dose MP treatment group and injury control group (8.1 ± 2.9 and 8.7 ± 1.1, respectively on day 7; 6.5 ± 2.2 and 6.4 ± 1.3, respectively on day 14) (Figure 3).

### Stress-dose hydrocortisone increased corticosteroid response and reduced incidence of critical illness-related corticosteroid insufficiency

TBI can result in acute activation of the HPA-axis and ultimately induce rapid secretion of glucocorticoids, norepinephrine, and inflammatory cytokines in order to strengthen a response to traumatic stress. We measured the concentration of CORT, the most abundant steroid released to the peripheral blood in injured rats, in response to ES. We have previously shown that levels of serum CORT increase rapidly after ES, peaking at 30 minutes, declining gradually and returning to baseline level by 24 h [13]. In the same study, we also demonstrated that the CII, the ratio of increased CORT to the baseline, is a reliable measure to evaluate the activation of the HPA axis. A CII value less than 2.5 was closely correlated with TBI mortality, and considered indicative of CIRCI as described previously [13].

**Figure 4:**
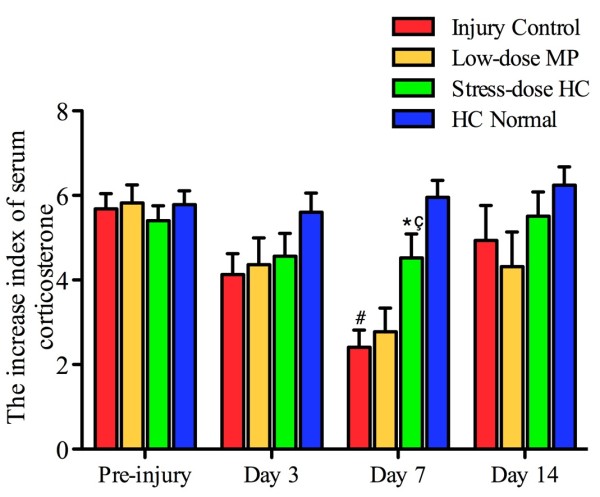
**Stress-dose hydrocortisone increases corticosteroid response after traumatic brain injury.** Corticosterone increase index (CII) of rats treated with stress-dose hydrocortisone (HC) was significantly higher than that of those from the injury control group or those treated with low-dose methylprednisolone (MP) on day 7 post-injury. Data are expressed as mean ± standard error of the mean. (^#^*P* <0.001 as compared to HC normal treatment; ^*^P = 0.005 as compared to injury control; ^ç^*P* = 0.022 as compared to low-dose MP). Pre-injury: n = 30 from each group; day 3: n = 30 from HC normal, n = 23 from injury control, n = 23 from low-dose MP, n = 26 from stress-dose HC; day 7: n = 30 from HC normal, n = 16 from injury control, n = 15 from low-dose MP, n = 22 from stress-dose HC; day 14: n = 30 from HC normal, n = 8 from injury control, n = 10 from low-dose MP, n = 17 from stress-dose HC.

CII, which was comparable among rats in different groups before injury, began to decrease on day 3, reached its lowest point on day 7, and returned to a level slightly below the baseline on day 14. It was significantly higher on day 7 in injured rats that received stress-dose HC (median 3.89, IQR 2.29 to 7.28) or low-dose MP (median 2.65, IQR 1.23 to 3.84) as compared to control rats (median 2.29, IQR 1.11 to 3.78) (Figure 4).

**Figure 5:**
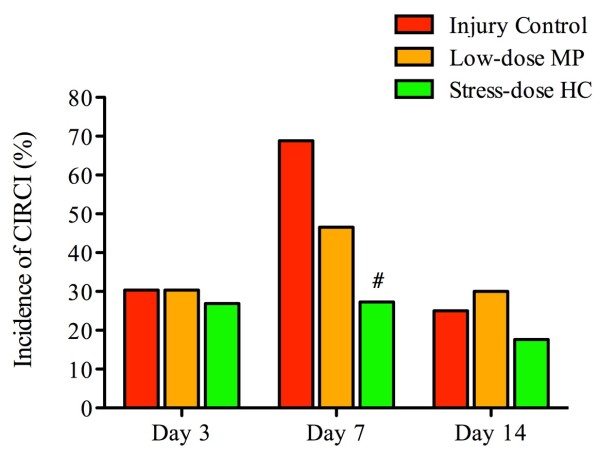
**Stress-dose hydrocortisone reduces the incidence of traumatic brain injury-induced critical illness-related corticosteroid insufficiency (CIRCI).** The incidence of CIRCI in rats treated with stress-dose hydrocortisone (HC) (27.3%) was significantly lower than in injury control rats (68.8%) and rats receiving low-dose methylprednisolone (MP) (46.6%) on post-injury day 7 (Pearson Chi-square = 6.446, df = 1, *P* = 0.011 as compared to injury control). However, a difference was not detected between stress-dose HC-treated rats and control animals on post-injury day 3 or 14. ^#^*P* = 0.02 as compared to injury control. Day 3: n = 23 from injury control, n = 23 from low-dose MP, n = 26 from stress-dose HC; Day 7: n = 16 from injury control, n = 15 from low-dose MP, n = 22 from stress-dose HC; day 14: n = 8 from injury control, n= 13 from low-dose MP, n = 17 from stress-dose HC.

Furthermore, the incidence of CIRCI in FPI rats treated with stress-dose HC (27.3%) was significantly lower than in those treated with saline (68.8%) or low-dose MP (46.6%) on post-injury day 7 (Figure 5). The reduction of CIRCI was paralleled with a decrease in TBI mortality (Pearson Chi-Square = 20.972, *P* <0.001) (Figures 2 and 5).

Together, these results suggest that (1) TBI resulted in acute CIRCI with a peak incidence on day 7 after injury; (2) stress-dose HC did not induce CIRCI in rats without injury; (3) stress-dose HC reduced the incidence of CIRCI after TBI, which may be a key factor for reducing mortality in rats with severe TBI.

### Stress-dose hydrocortisone reduced apoptosis in the paraventricular nucleii of the hypothalamus

TBI-induced corticosteroid response is initiated by signals from the PVN of the hypothalamus. Previous studies have shown that FPI causes neural apoptosis in this region [13]. Reducing the viability of cells in this region can negatively impact on the activation of the HPA axis. We therefore examined if the improvements on CII and CIRCI in rats treated with stress-dose HC is associated with reduced apoptosis in PVN cells.

**Figure 6:**
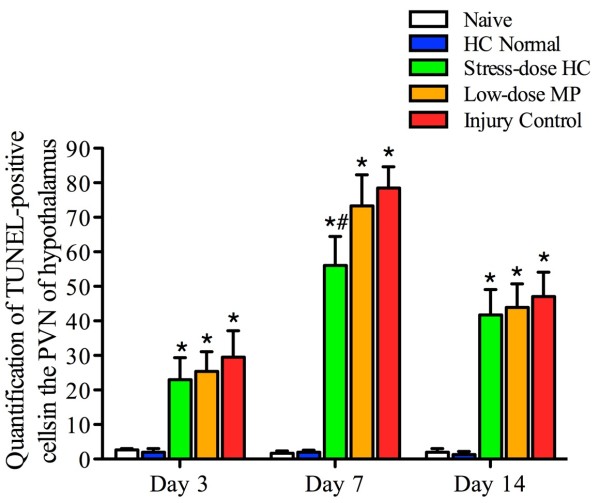
**Stress-dose hydrocortisone (HC) after traumatic brain injury decreases apoptosis.** Data represent the average number of terminal deoxynucleotidyl transferase dUTP nick end labeling (TUNEL)-positive cells in the paraventricular nuclei (PVN) area at 3, 7 and 14 days after injury, and are expressed as mean ± standard error of the mean. The number of TUNEL-positive cells was significantly higher in all injury groups compared to naïve rats, but they were significantly lower 7 days after injury in rats treated with stress-dose HC compared to injury controls. Analysis of variance, F_(4,31)_ = 11.589, *P* <0.001 for 7 days; post hoc least significant difference test, *P* = 0.041 for the stress-dose methylprednisolone (MP) group versus injury control. At each time point: n = 3 from each of the two groups (naïve and HC normal), n = 10 from each of three groups (injury control, low-dose MP, and stress-dose HC). ^#^*P* <0.05 as compared to injury control group; ^*^P <0.05 as compared to naïve group.

H&E staining showed that cells in the PVN of the hypothalamus became swollen and vacuolated after FPI. Over time, neurons shrank and became eosinophilic with pyknosistic nuclei. Three days after FPI, neuronal degeneration was observed, and both karyorrhexis and karyolysis were found in many PVN neurons and were most abundant on day 7 after injury. Figure 6 shows that TUNEL-positive cells significantly increased in the hypothalamus PVN of injured rats compared to non-injured rats. The numbers of TUNEL-labeled cells peaked 7 days after injury, when the incidence of CIRCI was also highest (Figures 5 and 6). Moreover, the number of TUNEL-positive cells was significantly reduced in rats receiving stress-dose HC (median 47.5, IQR 34.75 to 73.25) compared to injury control rats on post-injury day 7 (median 81.0, IQR 69.25 to 90.25).

### Stress-dose hydrocortisone reduced traumatic brain injury-associated vascular dysfunction

**Figure 7:**
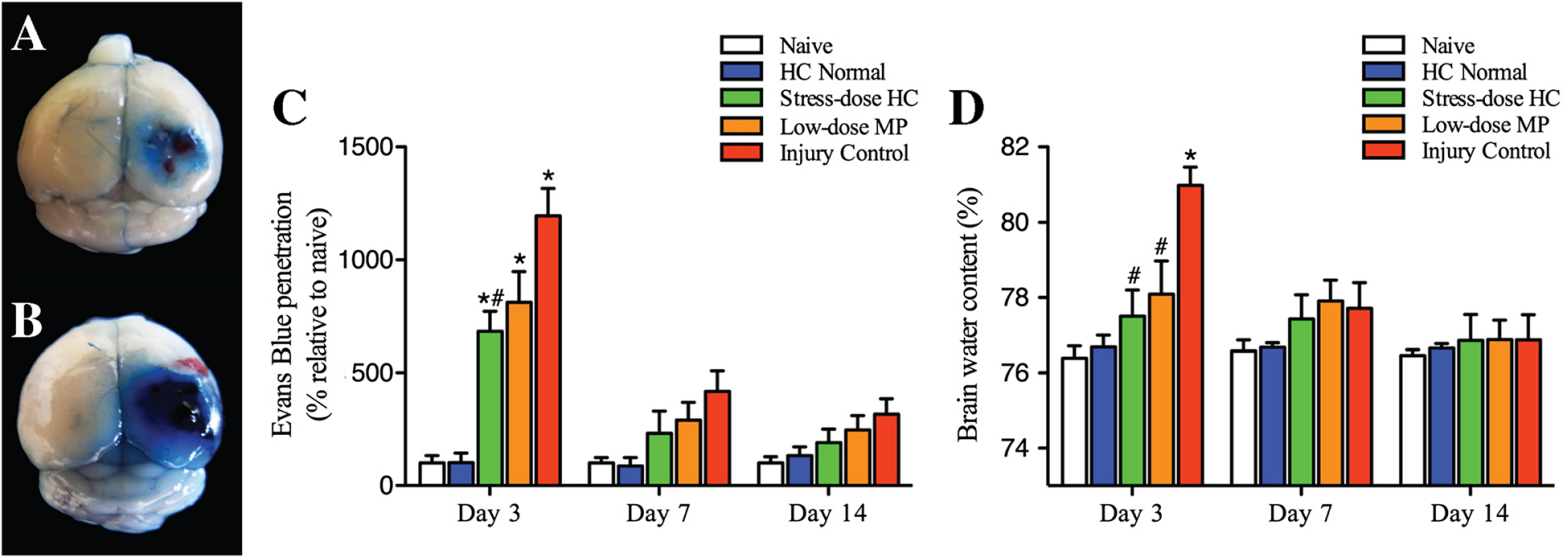
**Stress-dose hydrocortisone results in reduced blood–brain-barrier permeability in the hypothalamus.** Representative photographs of the brains from the stress-dose hydrocortisone (HC) group **(A)** and injury control group **(B)** and following Evans Blue (EB) dye injection on post-injury day 3. Summary data present the EB extravasation **(C)** and brain water content **(D)** in the hypothalamus at 3, 7 and 14 days after injury. The water content of the hypothalamus was significantly decreased in the stress-dose HC-treated rats as compared to injury controls (analysis of variance (ANOVA), F_(4,31)_ = 5.474, *P* = 0.002; post hoc least significant difference test (LSD), *P* = 0.001 for stress-dose HC versus injury control), and significant reduction in EB extravasation was also observed in the injured rats treated with stress-dose HC at 3 days after traumatic brain injury (ANOVA, F_(4,31)_ = 9.729, *P* = 0.000; post hoc LSD, *P* = 0.002 for stress-dose HC versus injury control). At each time point: n = 3 from each of the two groups (naïve and HC normal), n = 10 from each of three groups (injury control, low-dose methylprednisolone (MP), and stress-dose HC). ^*^P <0.05 as compared to naive; ^#^*P* <0.05 as compared to injury control. Data were expressed as mean ± standard error of the mean.

TBI compromises cerebral auto-regulation and breaks down the BBB, allowing blood cells and plasma infiltrate into the brain [33]. Several studies have shown that FPI causes an increase in vascular permeability, primarily in the ipsilateral hemisphere [30,34,35]. Figure 7C shows that TBI increased EB extravasation in the hypothalamus of injured rats as compared with control animals, and the post-injury administration of stress-dose HC significantly reduced this extravasation 3 days after injury. The same effects were also observed after low-dose MP administration, but to a lesser extent. No significant differences were observed among groups on either day 7 or 14 after injury. Figure 7D shows that water content in the hypothalamus increased significantly on post-injury day 3 and returned to pre-injury level on post-injury day 7. Both stress-dose HC and low-dose MP significantly reduced the water content 3 days after injury, but at different levels.

### Stress-dose hydrocortisone reduced traumatic brain injury-induced loss of endothelial cell and tight junction protein

**Figure 8:**
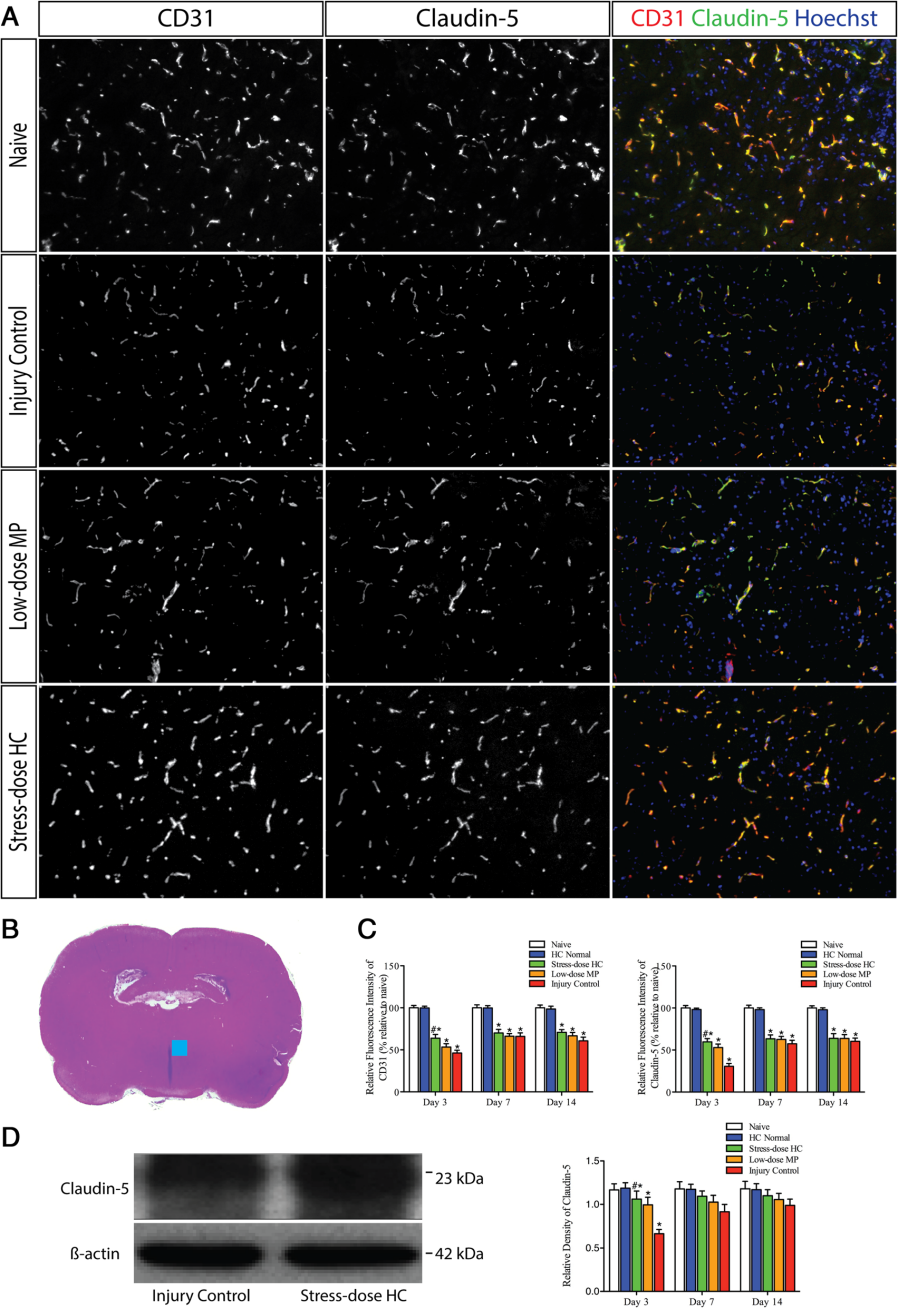
**Stress-dose hydrocortisone reduces the loss of CD31 and claudin-5 in the paraventricular nuclei (PVN) of the hypothalamus following trauamatic brain injury (TBI). (A)** Representative images of blood vessels in ipsilateral hypothalamus, immunostained for endothelial cell marker CD31, and tight junction protein Claudin-5. Stress-dose hydrocortisone (HC) appears to partially preserve loss of CD31 and claudin-5 caused by TBI, more pronounced with claudin-5. **(B)** Region of interest for immunoreactivity analysis was the PVN of the bilateral hypothalamus. Blue square indicates where the immunofluorescence pictures **(A)** were taken. **(C)** Summary data shows immunoreactivity of CD31 and claudin-5 were lowest on post-injury day 3, when significant BBB leakage was detected by EB extravasation measurement. Importantly, they were significantly increased by the administration of stress-dose HC, when overt improvement of BBB permeability was also observed. Analysis of variance (ANOVA), F_(4,45)_ = 54.625, *P* = 0.000 for CD31 comparison on day 3; post hoc least significant difference (LSD), *P* = 0.001 for stress-dose HC versus injury control, *P* = 0.000 for injury control versus naïve. F_(4,45)_ = 82.263, *P* = 0.000 for claudin-5 comparison; post hoc Dunnett T3, *P* = 0.000 for stress-dose HC versus injury control, *P* = 0.000 for injury control versus naive. **(D)** Representative western blot and summary data shows increased claudin-5 expression in the hypothalamus following administration of stress-dose HC on post-injury day 3. ANOVA, F_(4,31)_ = 5.716, *P* = 0.001; post hoc LSD, *P* = 0.001 for stress-dose HC versus injury control, *P* = 0.003 for injury control versus naive. At each time point: n = 3 from each of the two groups (naïve and HC normal), n = 10 from each of three groups (injury control, low-dose methylprednisolone (MP), and stress-dose HC). Data are mean ± standard error of the mean.

Figure 8A and B are representative immunofluorescence images of blood vessels labeled with CD31 and claudin-5 in the PVN of the ipsilateral hypothalamus. CD31, a receptor on endothelial junctions, was used to define the location of the blood vessel. Consistent with it being expressed at tight junctions of endothelium, claudin-5 is localized to the plasma membrane as a continuous strip-like staining pattern along the vasculature. Figure 8C shows that brain injury significantly decreased the immunoreactivity of CD31 and claudin-5 after injury. They were lowest on post-injury day 3, when significant BBB leakage was also detected by EB extravasation. Importantly, the immunoreactivity was significantly increased in the brain from rats treated with stress-dose HC, when overt improvement of BBB permeability was also observed. Stress-dose HC appears to protect the levels of claudin-5 expression, suggesting that the vascular integrity may have been preserved. Compared with a moderate increase in CD31 expression, increase in claudin-5 expression in the hypothalamus was more intensely detected by immunohistochemical staining and western blot, suggesting the preservation of tight junctions (Figure 8C and D).

## Discussion

In the present study, we examined changes in the corticosteroid response of rats to experimentally controlled severe FPI to the brain and correlated such changes to BBB dysfunction and neuronal apoptosis in the PVN of the hypothalamus. We compared these changes between rats receiving a 7-day treatment of stress-dose HC and those receiving low-dose MP. Our findings can be summarized as follows. First, 30 to 70% of injured rats developed CIRCI, peaking on post-injury day 7. The TBI-associated CIRCI was closely correlated with increased mortality and delayed neurological recovery. Second, post-injury treatment of rats with stress-dose HC reduced TBI-associated mortality and improved the recovery of neurological functions. Moreover, it increased corticosteroid response and prevented the development of CIRCI, which closely correlates with mortality. Third, stress-dose HC reduced neural apoptosis, preserved endothelial tight junction, and reduced BBB permeability in the PVN of the hypothalamus, starting on post-injury day 3. However these effects of stress-dose HC have not been observed in rats treated with low-dose MP.

There is increasing evidence to suggest that the dysfunction of the HPA axis occurs in a variety of critically ill patients, and is referred to as CIRCI [14,36]. Research and clinical interest has been increasingly focused on understanding the role of CIRCI in the pathological course of severe TBI. A recent retrospective clinical analysis suggests that CIRCI could develop in up to 50 to 70% of trauma patients with a mortality of 34% despite pharmacotherapy. To the best of our knowledge, no data on the spectrum of CIRCI in severe TBI are available to date. The detection of CIRCI in experimental animals has been difficult because there are no universally accepted diagnostic criteria [37]. We have previously described a set of criteria that can be used to define CIRCI in rats subjected to FPI based on an ES test that mimics acute stress, which activates the HPA axis in the sub-acute phase of TBI [13]. Using this platform, we found 30 to 70% of TBI rats developed CIRCI, with a peak incidence on post-injury day 7. The TBI-induced CIRCI was closely correlated with increased mortality and delayed neurological recovery.

Stress-dose HC, equivalent to the maximal endocrine secretion rate during critical illness, has been recommended for CIRCI patients with ARDS [2]. It has been reported to reduce death in these patients without increasing adverse events [38]. Emerging evidence suggests that this treatment could also reduce the occurrence of hospital-acquired pneumonia and the use of mechanical ventilation in patients with polytrauma and CIRCI [14]. Consistent with this, we found that post-injury administration of stress-dose HC increased the corticosteroid response and attenuated CIRCI following severe TBI. The restoration of the HPA axis function is closely associated with improved neurological outcomes and a reduced mortality.

Although the mechanism by which it efficiently reduces CIRCI-associated mortality is not completely known, the potent anti-inflammatory properties of stress-dose HC might enhance or accelerate the recovery of corticosteroid response to severe TBI. After initial injury, an injured brain is exposed to exaggerated inflammatory response and a large amount of cytokines activate the HPA axis. However, recent studies further suggest that excessive secretion of cytokines might cause CIRCI by suppressing the function of the HPA axis. The serum concentration of pr-inflammatory mediators, such as IL-6 and TNF-α, were shown to be markedly increased in CIRCI associated with septic shock [37] and TBI [39]. TNF-α has been shown to inhibit adrenocorticotrophic hormone (ACTH)-induced production of cortisol by suppressing the steroidogenic expression of P450 enzyme [40]. Stress-dose HC has been shown to efficiently suppress the production and activity of pro-inflammatory cytokines during TBI by inhibiting the nuclear factor (NF)-κB signaling pathway. This is in a sharp contrast to the high-dose glucocorticoid therapy that is reported to increase the risk of death in TBI [25,41-43].

In the present study, we showed that stress-dose HC reduced neuronal apoptosis in the PVN of the hypothalamus induced by TBI. This observation is consistent with previous studies by several laboratories, including our own [13,25,44]. By reducing apoptosis, stress-dose HC could prevent the disruption of corticotropin releasing hormone (CRH) transmission in the HPA axis and reduce CIRCI; both are prevalent in rats with severe TBI.

This anti-apoptotic effect could also explain why stress-dose HC improves vascular integrity as demonstrated by reducing the TBI-associated leakage of BBB. A disruption of BBB will allow the infiltration of circulating cells, plasma molecules, and fluid into the brain to exacerbate injury to the brain by FPI [45-48]. Our histological and immunochemical examinations of injured brain demonstrate that stress-dose HC increases molecules that are primarily expressed at the tight junctions of the vascular endothelium such as claudin-5. Our findings are consistent with previous reports that HC can maintain the integrity of tight junctions and thus, stabilize the BBB [20]. The activation of endogenous antioxidant proteins and inhibition of neuroinflammation restores claudin-5 levels and protects against TBI-induced increase in BBB permeability [49,50].

In contrast, we found low-dose MP neither reduced TBI-induced mortality nor improved the recovery of neurological function, which is the opposite of what we observed for treatment with stress-dose HC. Low-dose MP was also less effective in reducing apoptosis and improving BBB permeability in the PVN of the hypothalamus compared to stress-dose HC. One can speculate about the potential causes for this discrapany between the two gluococorticoids. First, HC has been shown to be four times more able to activate mineralocorticosteroid receptor (MR), but is four fifth less able to activate the glucocorticoid receptor (GR) compared to MP [51]. Recent studies also suggest that the difference in the rates of activation of MR and GR may account for this discrepancy [52]. For example, Chantong *et al.,* show that the balance between activated MR and GR is critical for neuronal excitability, stress responsiveness and behavioral adaptation. GR over-activation could modulate the activity of the excitatory and inhibitory neural inputs to the hypothalamic CRH neurons, and thus, block the stress-induced HPA activation in these neurons [53]. However, De Kloet *et al.,* suggest limbic MR activation maintains the basal HPA activity and the sensitivity or threshold of the central stress response system [54]. Furthermore, the activation of MR and GR has pro- and anti- apoptosis effects, depending on the type of target cells, including those critical for the integrity of the BBB [55-57].

The study is limited in its ability to determine whether the finding 1) can also be observed with female rats and is gender specific; 2) might be associated with change in partial arterial pressure (Pa)CO2, PaO2 and body temperature, which are key factors in the evolution of brain injuries, or the change in cytokine dosing in the brain or in the blood, which can directly influence hypothalamopituitary functions. Nevertheless, this study suggests that the improvement of BBB integrity and survival of hypothalamic neurons are two key activities that make stress-dose HC effective in improving neurological functions and reducing mortality in rats subjected to severe TBI.

## Conclusions

In summary, our findings add to the growing body of evidence supporting the use of stress-dose HC to improve outcomes of severe TBI. Stress-dose HC reduces CIRCI and improves neurological recovery, which is associated with reduction in damage to the tight junctions of vascular endothelial cells and neuronal apoptosis in the PVN of the hypothalamus. Therefore, translating this strategy to a clinical context may have potentially important therapeutic significance.

## Key messages

•The TBI-associated CIRCI is closely correlated with increased mortality and delayed neurological recovery. The peak incidence of CIRCI is on post-injury day 7

•Post-injury treatment with stress-dose HC reduces TBI-associated mortality and improves the recovery of neurological function

•Stress-dose HC treatment increases corticosteroid response and prevents the development of CIRCI, which closely correlates with reduction in mortality

•Stress-dose HC treatment reduces neural apoptosis, preserves endothelial tight junctions, and reduces BBB permeability in the PVN of hypothalamus

•Stress-dose HC can be considered as a potential treatment for TBI-induced CIRCI

## Abbreviations

ANOVA: Analysis of variance; ARDS: Acute respiratory distress syndrome; Atm: Atmosphere; BBB: Blood–brain-barrier; BWC: Brain water content; CII: Corticosterone increase index; CIRCI: Critical illness-related corticosteroid insufficiency; CORT: Corticosterone; EB: Evans blue; ELISA: Enzyme-linked immunosorbent assay; ES: Electrical stimulation; FPI: Fluid percussion injury; GR: Glucocorticoid receptor; H&E: Hematoxylin and eosin; HC: Hydrocortisone; HPA: Hypothalamus-pituitary-adrenal axis; i.p: Intraperitoneally; IL: Interleukin; LSD: Least significant difference; mNSS: Modified neurological severity score; MP: Methylprednisolone; MR: Mineralocorticosteroid receptor; PBS: Phosphate-buffered saline; PBST: Phosphate-buffered saline/Tween; PVN: Paraventricular nuclei; TBI: Traumatic brain injury; TNF-03B1: Tumor necrosis factor; TUNEL: Terminal deoxynucleotidyl transferase dUTP nick end labeling.

## Competing interests

The authors declare that they have no competing interests.

## Authors’ contributions

XC and JZ designed the experiment and wrote the manuscript. JD summarized, analyzed the data, and edited the manuscript. XC, ZZ, YC, LL and RJ performed the experiment. All authors read and approved the final manuscript.
